# Anti-inflammatory and antioxidative effects of six pentacyclic triterpenes isolated from the Mexican copal resin of *Bursera copallifera*

**DOI:** 10.1186/s12906-016-1397-1

**Published:** 2016-10-26

**Authors:** Antonio Romero-Estrada, Amalia Maldonado-Magaña, Judith González-Christen, Silvia Marquina Bahena, María Luisa Garduño-Ramírez, Verónica Rodríguez-López, Laura Alvarez

**Affiliations:** 1 Centro de Investigaciones Químicas-IICBA, Universidad Autónoma del Estado de Morelos, Avenida Universidad 1001, Chamilpa, Cuernavaca, Morelos 62209 Mexico; 2 Facultad de Farmacia, Universidad Autónoma del Estado de Morelos, Avenida Universidad 1001, Chamilpa, Cuernavaca, Morelos 62209 Mexico

**Keywords:** *B. copallifera*, Copal ancho, Pentacyclic triterpenes, Inflammation, NO inhibition, COX-2 inhibition

## Abstract

**Background:**

*Bursera copallifera* (Burseraceae) releases a resin known as “copal ancho” which has been used, since pre-Colombian times, as ceremonially burned incense and to treat tooth ache, tumors, arthritis, cold, cough, and various inflammatory conditions; however, its anti-inflammatory potential is poorly studied. The aim of the present study was to isolate, quantify, and to investigate the anti-inflammatory activity of triterpene compounds isolated from the copal resin of *B. copallifera*.

**Methods:**

The constituents present in the total resin of *B. copallifera* were obtained by successive chromatographic procedures, and quantitative analysis was performed by High Performance Liquid Chromatography (HPLC). Anti-inflammatory effects of the isolated triterpenes were investigated to determine their inhibitory effects on phorbol ester 12-*O*-tetradecanoylphorbol-13-acetate (TPA)-induced edema in mice, viability and nitric oxide (NO) production inhibition on lipopolysaccharide (LPS)-activated RAW 264.7 macrophages, and inhibition of cyclooxygenase (COX)-1, COX-2 and secretory Phospholipase A_2_ (sPLA_2_) activities in vitro.

**Results:**

Quantitative phytochemical analysis of the copal resin showed the presence of six pentacyclic triterpenes of which, 3-epilupeol (59.75 % yield) and α-amyrin (21.1 % yield) are the most abundant. Among the isolated triterpenes, 3-epilupeol formiate (Inhibitory Concentration 50 % (IC_50_) = 0.96 μmol), α.amyrin acetate (IC_50_ = 1.17 μmol), lupenone (IC_50_ = 1.05 μmol), and 3-epilupeol (IC_50_ = 0.83 μmol) showed marked inhibition of the edema induced by TPA in mice. α-amyrin acetate and 3-epilupeol acetate, at 70 μM, also inhibited the activity of COX-2 by 62.85 and 73.28 % respectively, while α-amyrin and 3-epilupeol were the best inhibitors of the production of NO in LPS-activated RAW 264.7 cells with IC_50_ values of 15.5 and 8.98 μM respectively, and did not affected its viability. All compounds moderately inhibited the activity of PLA_2_.

**Conclusions:**

This work supports the folk use of *B. copallifera* and provides the basis for future investigations about the therapeutic use of this resin in treating inflammation.

## Background

*Bursera* species are the dominant woody taxa in dry forests of México, where this genus reach its maximum diversity and abundance with about 84 species being present, 80 of which are endemic to the country [1–3]. These plants release a resin known as copal, derived from the Nahuatl language word “copalli” meaning incense [4]. This genus has been taxonomically related to *Commiphora* and *Boswellia*, which also produce resins known as myrrha and frankincense, respectively [5].

The resins obtained from *Bursera* spp. play an important role in the economy of rural families in México, and they are particularly identified with the aromatic resins used by the cultures of pre-Columbian Mesoamerica as ceremonially burned incense and other purposes. Copal is still used by a number of peoples of México and Central America as incense and during sweat lodge ceremonies, and the trees where the resins are obtained are today cultivated in many regions of México [4, 6]. Copal, as a traditional natural medicine, has been used to treat various diseases, such as tooth ache, tumors, fever, and inflammatory conditions. Tea made with the resin is a traditional remedy as analgesic and has been used to clean wounds and sores, and to cure bronchitis, cough and rheumatism since pre-Columbian time, and it is still used [7–9].

Among various resins collected by local people of Morelos state of México, “copal ancho” (*Bursera copallifera*, DC, Bullock) is considered as a source of high grade copal resin, and it is commonly used against rheumatoid arthritis, cold, cough, for stroke and dental pain, and for hasten wound healing [10, 11].

Previous studies have demonstrated the cytotoxic activity of the chloroform extracts obtained from the stems and fruits of *B. copallifera* [12]. More recently, our research team showed that the hydroalcoholic extract of the stems as well as the dichloromethane: methanol extract from the leaves inhibited the mouse ear inflammation in response to topical application of TPA by 54.3 and 55.4 % respectively, at the dose of 0.5 mg/ear [13]. Further, in this work, the mechanism for this anti-inflammatory effect was related to the direct inhibition of COX-1 and moderate of COX-2, which are associated with inflammatory diseases. However, the anti-inflammatory potential of the resin and its constituents are still unknown.

The ethnomedicinal importance of *B. copallifera* and its components, prompted us to undertake detailed investigation on the constituents of the resin and their anti-inflammatory activity in order to evaluate its anti-inflammatory potential and compare with those described for the other parts of the plant. Although the TPA‑induced mouse ear model of inflammation is nonspecific, it is widely used for acute anti‑inflammatory screening because TPA activates PLA_2_, [14] and the resulting edema is primarily mediated by prostaglandin E2 (PGE2) [15]. Thus, both PLA_2_ and COX are involved in this model, and it has been demonstrated that the organic extracts of *B. copallifera* interfere with these enzymes to inhibit TPA‑induced inflammation.

In this paper, we report the isolation and identification of six triterpenes (**1**–**6**) with anti-inflammatory activity, isolated from the *n*-hexane soluble fraction of the resin of *B. copallifera*. In addition, quantification of the active components in the resin was also performed by HPLC analysis. The anti-inflammatory activity of the six isolated compounds was evaluated in the mice model of TPA-induced edema. Furthermore, we examined the inhibitory effects of these triterpenes on cell viability and NO production in LPS- stimulated RAW 264.7 macrophages. Also, their inhibitory effects over COX-1, COX-2, and *s*PLA_2_ activities in vitro were assayed.

## Methods

### Materials and reagents

Silica gel (70–230 mesh, ASTM and 230–400 mesh) and Preparative Thin Layer Chromatography (TLC) were purchased from Merck. Deuterated chloroform (CDCl_3_), TPA, indomethacin, LPS from *Escherichia coli* serotype 055:B5, sodium nitrite (NaNO_2_), N-(1-naphtyl) ethylenediamine dihydrochloride and sulfanilamide were purchased from Sigma Aldrich. Dulbecco’s Modified Eagle’s Medium/Nutrient Mixture F-12 (DMEM/F12), fetal bovine serum (FBS) and Glutamine (GlutaMax) were from GIBCO, [3-(4,5-dimethyl-2-yl)-5-(3-carboxymethoxyphenyl)-2-(4-sulfophenyl)-2H-tetrazolium, inner salt; MTS] was from Promega Co. *s*PLA_2_, COX-1 and COX-2 ELISA kits were purchased from Cayman Chemical Co.

### Plant material

The resin of *B. copallifera* (DC.) Bullock was collected in August 2011 at El limón de Cuahuchichinola (N 18° 31′ 16.5”), in the Reserva de la Biósfera Sierra de Huautla (REBIOSH) by M. C. Teresita Rodríguez López. Voucher specimen No. 31809 was deposited at the Herbarium of the University of Morelos (HUMO) in the Centro de Investigación en Biodiversidad y Conservación (CIByC) at the Universidad Autónoma del Estado de Morelos (UAEM).

### Compound isolation

The resin of *B. copallifera* was air-dried at room temperature for 4 weeks, ground and homogenized to an uniform powder by ceramic mortar with pestle. 20 g of the resin powder was totally dissolved with 50 mL of a mixture of dichloromethane:acetone (9:1) at room temperature and subjected to column chromatography (CC) on 150 g silica gel (70–230 mesh, ASTM), and stepwise gradient elution with *n*-hexane:acetone (1:0 → 1:1, v/v). Three fractions of 1.5 L each were collected, *n*-hexane (F-1, 6.06 g), *n*-hexane:acetone 9:1 (F-2, 8.8 g) and *n*-hexane:acetone 8:2 (F-3, 5.1 g).

**Fig. 1:**
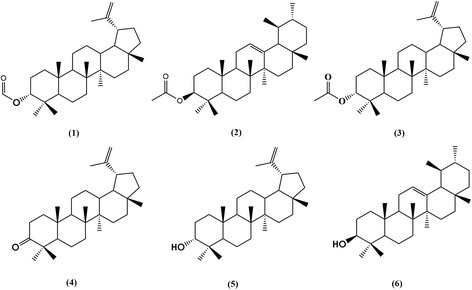
Triterpenes isolated from the resin of *Bursera copallifera*

F-1 was subjected to CC on 165 g silica gel (230–400 mesh) using a mixture of hexane:dichloromethane 95:5 as the isocratic eluent, 100 mL fractions were collected through-out and pooled into four groups (F1-1 to F1-4) according to composition, as visualized by TLC. F1-1 (78.2 mg) was purified by preparative TLC (n-hexane:dichloromethane 93:7) to afford 45.9 mg of lup-20(29)-en-3α-ol formiate (**1**) and 9.6 mg of ursan-3β-ol acetate (**2**), F1-2 (271.2 mg) afforded 245 mg of lup-20(29)-en-3α-ol acetate (**3**) after acetone crystallization. F1-3 (5.4 g) was subjected to CC on silica gel (230–400 mesh), using *n*-hexane:Ethyl acetate gradient (9:1 → 1:1) which gave three major fractions, F1-3A (2.66 g), F1-3B (115.1 mg) and F1-3C (2.61 g). F1-3A was purified by silica gel (230–400 mesh) chromatographic column eluting with an isocratic mixture of *n*-hexane:dichloromethane (95:5) to afford 8.4 mg of lup-20(29)-en-3-one (**4**), and 2.2 g of lup-20(29)-en-3α-ol (**5**). Fraction F1-3B gave pure **5** (115.1 mg), and fraction F1-3C was purified by silica gel (230–400 mesh) CC, using *n*-hexane:Ethyl acetate (98:2) to afforded 2.5 mg of lup-20(29)-en-3α-ol (**5**) and 0.0122 mg of pure α-amyrin (**6**). F-2 was constituted for lup-20(29)-en-3α-ol (**5**) and α-amyrin (**6**) principally. The structures of isolated compounds were identified by spectroscopic and spectrometric analyses (Fig. 1). The samples were dissolved with CDCl_3_ and Nuclear Magnetic Resonance (NMR) spectra were acquired on a Varian Unity NMR spectrometer operating at 400 MHz for ^1^H and 100 MHz for ^13^C nuclei. For the structural determination, the experiments ^1^H–^1^H gCOSY, NOESY, gHSQC and gHMBC were analyzed as required. FABMS spectra were recorded on a JOEL JMX-AX 505 HA mass spectrometer.

### Quantitative analysis by HPLC

Resin dried and pulverized (3 mg) and standards (3 mg) were prepared by sonicating (10 min) in methanol (1 mL) and analyzed by HPLC. All samples were filtered through 0.45 μm syringe filter and injected into HPLC. Chromatography was carried out on a Waters 600E gradient module HPLC system, Waters 717 plus Autosampler, waters 996 photodiode array detector and computer with EmpowerPro of waters. The column used was a reversed phase Chromolith SpeedROD RP-18e (50 mm × 4.6 mm), from Merck. The separation was carried out isocratically using Acetonitrile:Water (95:05) as the mobile phase (40 min). The system was operated at room temperature and monitored at 210 nm. The flow rate was 0.5 mL/min and the injection volume was 20 μL. The standard samples of lup-20(29)-en-3α-ol formiate (**1**), ursan-3β-ol acetate (**2**), lup-20(29)-en-3α-ol acetate (**3**), lup-20(29)-en-3-one (**4**), lup-20(29)-en-3α-ol (**5**) and α-amyrin (**6**) compounds were isolated from the resin of *B. copallifera* and characterized in our laboratory. The purity (>98) of the isolated compounds was confirmed by HPLC and ^1^H NMR analysis. Quantification was performed by comparing their retention times with the standards and calculating the concentration from the respective calibration curves. The assay was performed in triplicate.

### In vivo anti-inflammatory activity

#### TPA-induced mouse ear edema

Mouse ear edema was evaluated following the described protocol [16]. All experiments were carried out using six animals per treatment. Adult male CD-1 mice with a body weight ranging from 25 to 30 g were used. Experiments were performed according to the Official Mexican Rule: NOM-062-ZOO-1999 Guidelines (Technical Specifications for the Production, Care, and Use of Laboratory Animals) and international ethical guidelines for the care and use of experimental animals. The experimental protocol followed was approved by Comité de Experimentación del Bioterio of the Universidad Autónoma del Estado de Morelos (BIO-UAEM) (Approval number: BIO-UAEM: 009:2013). Mice were maintained under standard laboratory conditions (Bioterio at the Universidad Autónoma del Estado de Morelos) at 22 °C ± 3 °C, 70 % ± 5 % of humidity, 12 h light/dark cycle and food/water *ad libitum*. A negative control group received acetone as vehicle and indomethacin was used as anti-inflammatory drug as positive control group. Finally, compounds were tested by separate treatment groups. Animal ear inflammation was induced with 2.5 μg of TPA dissolved in 20 μL of acetone applied to the internal and external surface of the right ear to cause edema. Sample doses of 1, 0.75, 0.50, 0.25 and 0.125 mg/ear of the compounds, as well as the anti-inflammatory drug of reference (indomethacin) were applied. All the samples of the different treatments were dissolved in (20 μL of acetone or ethanol) depending on the solubility of the specified compound and applied topically on the right ear immediately after TPA application; on the left ear acetone or ethanol was applied as vehicle. Four hours after application of the samples of interest as possible anti-inflammatory agents, the animals of each treatment were sacrificed by cervical dislocation. Circular sections of 6 mm in diameter were taken from both: the treated (t) and the non-treated (nt) ears, which were weighed to determine the inflammation. Percentage of inhibition was determined by the formula expressed below:

$$ Inhibition\ \%=\left(\varDelta w\  control - \frac{\varDelta w\  treatment}{\varDelta w}\right)\times 100 $$

where Δw = w_t_ − w_nt_; being w_t_ the weight of the section of the treated ear and w_nt_ the weight of the section of the non-treated ear. The IC_50_ values of the anti-inflammatory activity obtained at the doses of 1, 0.75, 0.50, 0.25 and 0.125 mg/ear were calculated using GraphPad Prism® software by lineal regression analysis.

### In vitro anti-inflammatory activities

#### Cell culture

Murine macrophage cell line RAW 264.7 (Tib-71tm from ATCC) were grown in DMEM/F12 medium supplemented with 7.5 % heat-inactivated FBS, GlutaMax, without antibiotics. Cells were plated and incubated in a humidified atmosphere containing 5 % CO_2_ at 37 °C. Cells were sub-cultured by scraping and seeding them in 75 cm^2^ flasks or 24-wells plates.

### Treatment of macrophages with LPS

RAW 264.7 cells (1.4 × 10^5^ cells/well) were plated and incubated into 24-well plates in 0.5 mL of DMEM/F12 medium supplemented with 7.5 % heat-inactivated FBS, for 24 h, at 37 °C with 5 % CO_2_. After that, macrophages were incubated for two hours with the test compounds (**2**–**6**) at various concentrations (0–70 μM) or vehicle (Dimethyl sulfoxide (DMSO), 0.5 %, v/v) or indomethacin (30 μg/mL). Then, macrophages were incubated with LPS (10 μg/mL) in the presence or absence of test compounds, indomethacin or vehicle and without LPS at 37 °C for 20 h to stimulate NO production. Finally, cell-free supernatants were collected and were kept at -20 °C until NO quantification. The suppressive effect of compounds **2–6** on NO production was assessed using the Griess reagent

### Determination of NO concentration

Nitrite, the stable end product of NO, was used as an indicator of NO production in the culture medium. Nitrite released in the culture medium was measured according to Griess reaction. Briefly, 50 μL of each cell culture supernatants were mixed with 100 μL of Griess reagent (50 μL of 1% sulfanilamide and 50 μL of 0.1% N-(1-naphtyl) ethylenediamine dihydrochloride in 2.5 % phosphoric acid), for 10 min at room temperature. The optical density at 540 nm (OD_540_) was measured with a microplate reader and nitrite concentration in the samples were calculated by comparison with the OD_540_ of a standard curve of NaNO_2_ prepared in fresh culture medium [17].

### MTS-tetrazolium salt assay

Cell viability was measured based on the formation of blue formazan metabolized from colorless MTS by mitochondrial dehydrogenases, which are active only in live cells. RAW 264.7 macrophages were plated in 96-well plates at a density of 1.2 × 10^4^ cells per well for 24 h. The cells were treated and incubated with various concentrations of test compounds (0–70 μM) for 24 h. Cell viability was determined by MTS assay, using the CellTiter 96 Aqueous Non-Radioactive Cell Proliferation assay (Promega). Briefly, 20 μl of MTS was added to each well, and the cells were incubated for another 4 h at 37 °C with 5 % CO_2_. The optical density was measured at 490 nm on a microplate reader.

### *s*PLA_2_ enzyme inhibitory assay

Activity of *sP*LA_2_ was evaluated by the test described in the *s*PLA_2_ (Type V) Inhibitor screening Assay kit No. 10004883 from Cayman Chemical Co., according to the manufacturer’s instructions. The compounds (**1–6**) at 70 μM and positive control palmitoyltrifluoromethylketone at 4 μM dissolved in DMSO or ethanol were assayed.

### COX-1 and 2 enzyme inhibitory assay

All compounds described here were tested for their ability to inhibit COX-1 and COX-2 using a COX-1(ovine) and COX-2 (human)-inhibitor screening assay kit No. 701050 from Cayman Chemical Co., according to the manufacturer’s instructions. The compounds (**1–6**) at 70 μM and selective COX-1 inhibitor, SC-560 (3.3 μM) and selective COX-2 inhibitor, DuP-697 (3 μM) dissolved in DMSO or ethanol were assayed.

### Statistical analysis

The results shown were obtained at least by three independent experiments and are presented as means ± SDs. Statistical analyses were performed by one-way analysis of variance (ANOVA) with Tukey’s post hoc test. All statistical analyses were performed using the OriginLab (Massachustts USA), version 8.0 software. *P* values ˂ 0.05 were considered to indicate statistical significance.

## Results

### In vivo anti-inflammatory activity of the resin

The resin of *B. copallifera* was dissolved with a mixture of dichloromethane:acetone (8:2) at room temperature, this extract showed inhibition on TPA-induced auricular edema in mice (50% inhibitory dose (ID_50_) = 0.7071 mg/ear).

### Triterpenes isolation

Repeated silica gel column chromatography of the active extract, allowed the isolation of six bioactive triterpenes. The structures of compounds **1**–**6** (Fig. 1) were determined using ^1^H, ^13^C NMR, and Mass Spectrometry (MS) data which were in complete agreement with reported ones, 3-epilupeol formiate (**1**), α-amyrin acetate (**2**), 3-epilupeol acetate (**3**), lupenone (**4**), 3-epilupeol (**5**) and α-amyrin (**6**) [18–24].

### Quantitative analysis of triterpenes by HPLC

HPLC quantification of the triterpenes present in the total resin was performed using authentic 3-epilupeol formiate (**1**), α-amyrin acetate (**2**), **3**-epilupeol acetate (**3**), lupenone (**4**), 3-epilupeol (**5**), and α-amyrin (**6**) as standards. The results showed that 3-epilupeol (**5**, 59.75 %) and α-amyrin (**6**, 21.1 %) are the most abundant triterpenes in the resin. The minor triterpenes were α-amyrin acetate (**2**, 6.25 %), 3-epilupeol acetate (**3**, 11.31 %), lupenone (4, 1.82 %), and 3-epilupeol formiate (**1**, 0.5 %).

### In vivo anti-inflammatory activity of triterpenes

The isolated triterpenes **1**–**6** were evaluated at different concentrations in TPA-induced auricular edema in mouse model. Table 1 illustrates the anti-inflammatory activity displayed by these compounds. Except for compounds **3** and **6**, all the triterpenes showed marked anti-inflammatory activity with ID_50_ values ranging from 0.83 to 1.17 μmol.

**Table 1:** Effect produced by resin and pure compounds 1–6 from *B. copallifera* on auricular edema induced by TPA in mice

	Edema inhibition (%) (1 mg/ear)	ID_50_ μmol/ear
Total extract from *B. copallifera* resin	55.14 ± 3.85	0.707^a^
3-epilupeol formiate (**1**)	62.16 ± 1.80	0.96
α-amyrin acetate (**2**)	69.45 ± 0.87	1.17
3-epilupeol acetate (**3**)	49.35 ± 3.6	>2.13
Lupenone (**4**)	57.25 ± 1.36	1.052
3-epilupeol (**5**)	66.39 ± 4.38	0.83
α-amyrin (**6**)	25.00 ± 1.81	>2.34
Indomethacin	91.35 ± 0.47	0.67

Data represent the mean ± S.D. of at least three independent experiments performed in triplicate. ^a^The dose was in mg/ear

### In vitro anti-inflammatory activity of triterpenes

**Fig. 2:**
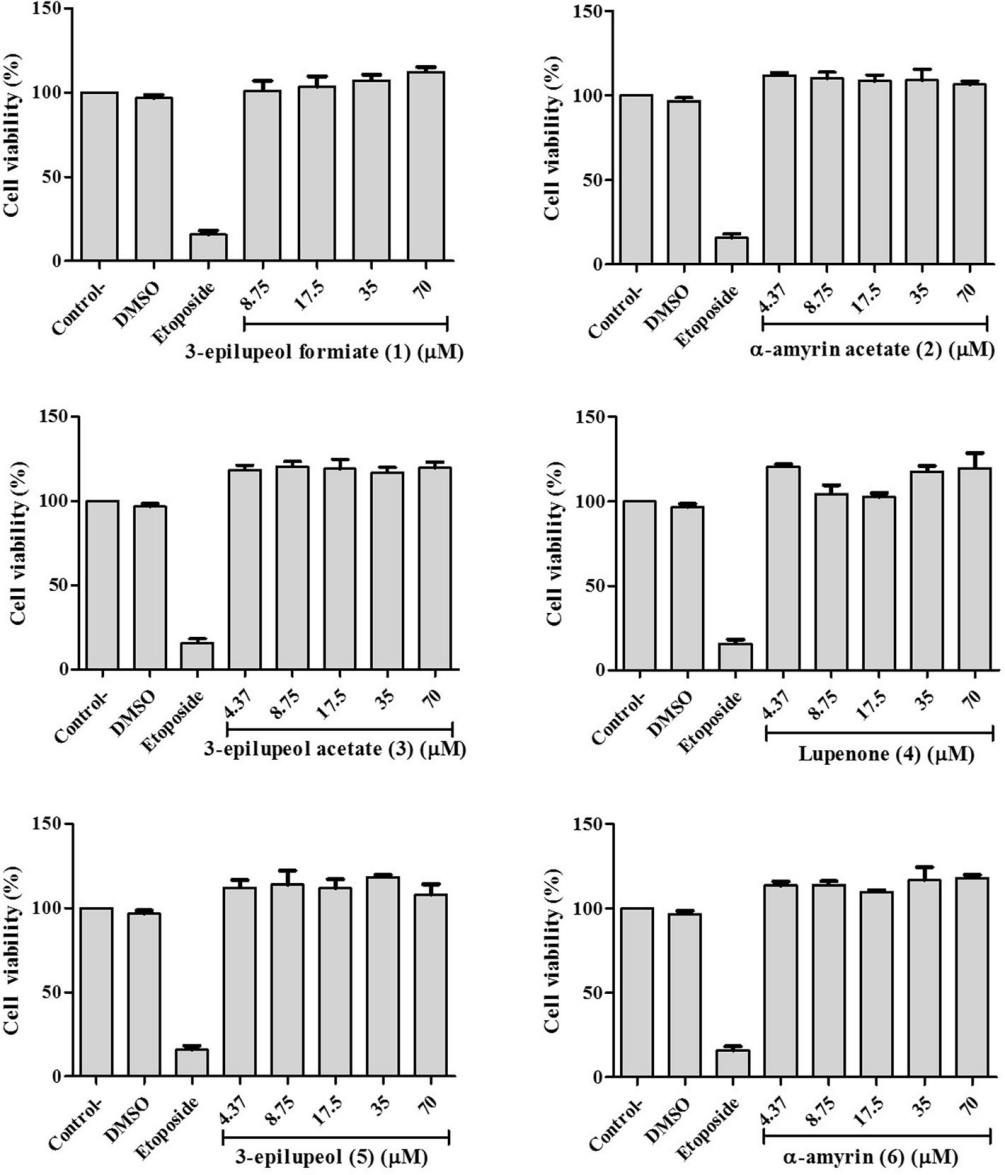
Effect of natural triterpenes **1**–**6** on the viability of LPS-stimulated RAW 264.7 cells. The values are expressed as the mean ± SD of three independent experiments

The effects of triterpenes **1**–**6** on the viability of the RAW 264.7 cells was determinated at different concentrations (4.37, 8.75, 17.5, 35.0, and 70.0 μM) (Fig. 2), and all the triterpenes did not exhibit a significant reduction in viability of macrophages compared con the positive control, up to the concentration of 70 μM.

**Fig. 3:**
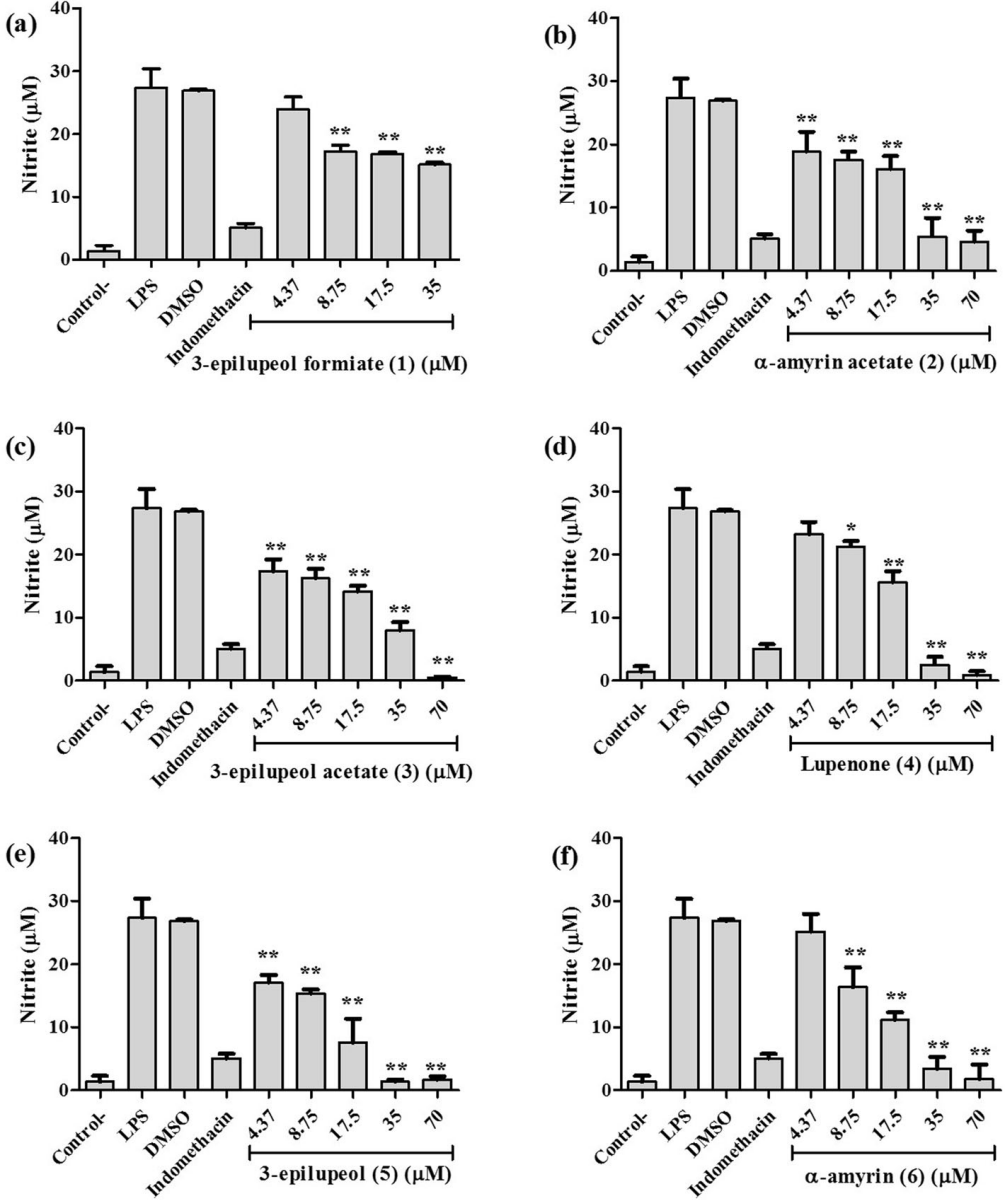
Effect of the isolated triterpenes on NO∙production in LPS-stimulated RAW 264.7 cells. (**a**) 3-epilupeol formiate, (**b**) α-amyrin acetate, (**c**) 3-epilupeol acetate, (**d**) lupenone, (**e**) 3-epilupeol, and (**f**) α-amyrin. The nitrite values are the mean ± SD from three independent experiments. Significance was determined by one-way ANOVA (*$P$ < 0.01; ** $P$ < 0.001compared to LPS)

To assess the effect of the triterpenes (**1**–**6**) isolated from *B. copallifera* resin on production of NO in LPS-induced RAW 264.7 cells, cells were treated with/without natural products (4.37, 8.75, 17.5, 35.0, and 70.0 μM) for 2 h and then stimulated with LPS (10 μg/ml) for 24 h. The amount of nitrite, a stable metabolite of NO, was used as the indicator of NO production in the medium. Among the isolated triterpenes, it has been described that lupenone (**4**) reduced NO production with an IC_50_ value of 10.81 μM, in LPS-stimulated RAW 264.7 cells [25], and was included as the positive control. The experimental results showed that NO level was increased in LPS-stimulated RAW cells, and this effect was decreased significantly by treatment with compounds **1**–**6** (*P* < 0.001) (Fig. 3). The IC_50_ values are gathered in Table 2 and it can be seen that α-amyrin (**6**) was the most active compound with IC_50_ value of 8.98 μM, while 3-epilupeol formiate (**1**) was the less active one with IC_50_ value of 43.31 μM.

**Table 2:** Effect of the natural triterpenes **1**–**6** on NO production on RAW 264.7 macrophages

Compound	NO^a^ IC_50_ (μM)
3-epilupeol formiate (**1**)	43.31 ± 2.60
α-amyrin acetate (**2**)	22.57 ± 1.19
3-epilupeol acetate (**3**)	31.13 ± 1.25
Lupenone (**4**)	20.80 ± 1.07
3-epilupeol (**5**)	15.50 ± 1.14
α-amyrin (**6**)	8.98 ± 1.73
Indomethacin (83.8°μM)	54.69 ± 10.34

^a^Data represent the means ± SD (*n* = 3). Values are an average of three independent experiments performed in triplicate (*p* < 0.001)

Evaluation of the inhibition of the enzymes COX-1, COX-2 and PLA_2_ showed that neither of the natural triterpenes inhibited COX-1 when evaluated at 70 μM, and only compounds **2** and **3** inhibited by 62.85 % and 73.28 % respectively the activity of COX-2 at 70 μM. Evaluation of the inhibition of PLA_2_ showed that all compounds inhibited moderately this enzyme **2** (17.27 %), **3** (12.6 %), **4** (7.12 %), **5** (16.6 %), and **6** (9.31 %) at 70 μM.

## Discussion

The resin of *B. copallifera*, known as “copal ancho”, has been used to treat various inflammatory diseases [10, 11, 13]. Despite the importance of this plant species, there is little knowledge about the anti-inflammatory activity and the potential anti-inflammatory components. In this work, we demonstrated that the total resin of *B. copallifera* inhibited the TPA-induced edema on mice with ID_50_ value of 0.7071 mg/ear. Phytochemical analysis of this resin allowed the isolation of six triterpene compounds which were characterized as 3-epilupeol formiate (**1**), α-amyrin acetate (**2**), 3-epilupeol acetate (**3**), lupenone (**4**), 3-epilupeol (**5**) and α-amyrin (**6**). Pentacyclic triterpenes are commun metabolites in the resins of *Bursera* species. *B. delpechiana* contains principally triterpenes with ursan skeleton, including α-amyrin [26]; the stem of *B. graveolens* contain lignans, and the triterpenes lupeol and epilupeol [27], and the leaves produce flavonoids and the triterpene α-amyrin [28]; *B. simaruba*, synthesize lupene-related pentacyclic triterpenes such as lup-20(29)-en-3β,23-diol, lupeol, epilupeol, epiglutinol and α-amyrin [29–31]. Finally, *B. microphylla* was reported to have malabaricane type triterpenes [32]. Previous studies had already described the existence of lupeol and lupenone on the resin of *B. copallifera*, collected in Guerrero state, México [33], but this is the first report on the presence of triterpenes **1**–**3** and **5**–**6** in the resin of *B. copallifera*, where 3-epilupeol (59.75 %) and α-amyrin (21.1 %) were identified as the major components. The high yields of **5** and **6** are in accordance with those described for the commercial Mexican copal Sonora resin in where these triterpenes are in 73 and 21 % yields respectively [34].

Among the isolated triterpenes, in recent years α-amyrin (**6**) has attracted much interest because its multiple pharmacological effects, principally as antinociceptive, anti-inflammatory, antipruritic, hepatoprotective, antihyperglycemic, and it has been demonstrated that the topical anti-inflammatory activity involve the inhibition of PGE2 level via inhibition of the COX-2 expression [35, 36]. Epilupeol (**5**), however, has been less studied, although its anti-inflammatory [37], antitubercular [38], and cytotoxic [39] activities have been described. 3-epilupeol acetate (**3**) has been reported to have α-glucosidase inhibitory activity [40]. 3-epilupeol formiate (**1**), was reported as constituent of *Boswellia carterii* [18], and until now there is not reports about its biological activity.

Except for compounds **3** and **6**, all the triterpenes showed marked anti-inflammatory activity when tested in the TPA-induced ear edema in mice. Triterpenes with the lupane skeleton showed the best activity, being 3-epilupeol (**5**, ID_50_ = 0.83 μmol/ear), together with its 3-formyl ester (**1**, ID_50_ = 0.96 μmol/ear) the most active compounds. 3-epilupeol acetate (**3**) (ID_50_ = > 2.13 μmol), and α-amyrin (**6**) (ID_50_ = > 2.34 μmol) were the less active. In contrast, α-amyrin acetate (**2**) was active with ID_50_ value of 1.17 μmol/ear. A survey of the literature, about the anti-inflammatory properties of the isolated compounds, revealed that compounds **2**, **4**, **5** and **6** were previously evaluated in the TPA-induced edema in mice [37, 41–44]. The results obtained in this work matched well with those described, except for α-amyrin (**6**) probably because its poor solubility.

Further, we evaluated the effects of these natural triterpenes on the production of NO in RAW 264.7 macrophages. For comparison, the activity of lupenone (**4**) was included as positive control. The cytotoxicities of compounds in RAW 264.7 cells were also assessed using MTS assay [45]. In all cases, the natural triterpenes exhibited potent NO production inhibitory activities, and did not affect the cell viabilities in either the presence or absence of LPS, even at a concentration of 70 μM, indicating no significant effect of exposure of the cells to LPS at the concentrations used (Fig. 2). The results indicate that all compounds are effective inhibitors of LPS-induced NO production in these cells. Indeed, as is shown in Fig. 3, the production of NO was markedly elevated in response of LPS. However, the application of the triterpenes **1**–**6** inhibited the production of NO by LPS in a concentration-dependent manner, and as shown in Table 2, compounds **2**, **4**, **5** and **6** exhibited inhibitory potency with IC_50_ values of 22.47, 20.8, 15.5 and 8.98 μM, respectively. Compounds **1** and **3** displayed moderate effects with IC_50_ values of 43.31 and 31.13 μM, respectively. Although lupenone (**4**) inhibited NO production at 10.81 μM in the previous report [25], the IC_50_ value obtained in our assay (20.8 μM) was of equivalent order of magnitude.

3-epilupeol (**5**) (IC_50_ = 15.5 μM) showed higher potency than lupeol (IC_50_ = 64.65 μM) isolated from *Pueraria lobata* roots [25], and lesser than the lanostan-type triterpene butyl lucidenate Q (IC_50_ = 4.3 μM), isolated from *Ganoderma lucidum* [46]. The best inhibitor was α-amyrin with IC_50_ value of 8. 98 μM, better than that displayed by ursolic acid with 9.3 % NO production inhibition with 43.8 % cell viability at 10 μM [47].

Evaluation of the inhibition in vitro of the enzymes COX-1, COX-2 and PLA_2_ activities showed that neither of the natural triterpenes inhibited COX-1 when evaluated at 70 μM, and only compounds **2** and **3** inhibited by 62.85 % and 73.28 % respectively the activity of COX-2 at 70 μM. 3-epilupeol (**3**) showed the highest inhibition of COX-2. Lupenone (**4**) has been described that inhibit the activity of COX-2 by 40 % at 100 μg/mL [48], while in our assay, lupenone (**4**) displayed 9.8 % inhibition at 70 μM. The inhibitory effects of these compounds were compared with selective COX-1 inhibitor, SC-560 (90% inhibition at 3.3 μM) and selective COX-2 inhibitor, DuP-697 (>90 % inhibition at 3 μM) provided by the kit assay. Evaluation of the inhibition of PLA_2_ showed that all compounds inhibited moderately this enzyme **2** (17.27 %), **3** (12.6 %), **4** (7.12 %), **5** (16.6 %), and **6** (9.31 %) at 70 μM, while the positive control palmitoyltrifluoromethylketone produced 25 % inhibition of PLA_2_ at a concentration of 4 μM [13].

Accumulating evidence has indicated that NO is well known for its involvement in the development of inflammation [49, 50]. NO is an important intra- and intercellular signaling molecule in cardiovascular, nervous, and immunological systems. NO is involved in various biological reactions including vasorelaxation, inhibition of platelet aggregation, neurotransmission, inflammation, and immunoregulation [51, 52]. Therefore, identifying new agents capable of lowering the production of this proinflammatory agent is regarded as an essential requirement for the alleviation of a number of inflammation-related disorders attributed to macrophage activation [53]. Similarly, COX and PLA_2_ are key enzymes in the synthesis of inflammatory prostaglandins which contributes to pathogenesis of various inflammatory diseases, edema, angiogenesis, invasion, and growth of tumor. COX-1 is a constitutively expressed enzyme with general housekeeping functions. COX-2 is an inducible enzyme that catalyzes biosynthesis of PGE2 [54, 55]. PLA_2_ catalyze the hydrolysis of the phospholipid sn-2 ester bond, generating a free fatty acid and a lysophospholipid. The PLA_2_ reaction is the primary pathway through which arachidonic acid (AA) is liberated from phospholipids. Free AA is the precursor of the eicosanoids, which include the prostaglandins, generated through the COX reaction, and the leukotrienes, generated through the lipoxygenase reaction [56].

## Conclusions

In conclusion the total resin of *B. copallifera* possess significant and promising anti-inflammatory activity. In this study, we showed that in LPS-stimulated macrophages, the isolated compounds **1**–**6** dose-dependently inhibited NO and triterpenes α-amyrin acetate (**2**) and 3-epilupeol acetate (**3**) inhibited the activity of COX-2, while all of them showed moderate inhibitory activity of PLA_2_ enzyme, suggesting that this was the mechanism underlying the observed anti-inflammatory activity observed in vivo.

The study also signifies that isolated constituents could be responsible, at least in part, for its anti-inflammatory activity. The study verifies traditional use of *B. copallifera* for the treatment of rheumatism, asthma, and other inflammatory disorders.

## Abbreviations

%: Percentage
^13^C: 13-carbon isotope
^1^H: Proton
^1^H–^1^H gCOSY: Gradient-selected homonuclear correlation spectroscopy
ANOVA: Analysis of variance
CDCl_3_: Deuterated chloroform
CH_2_Cl_2_: Dichloromethane
CH_3_CN:H_2_O: Acetonitrile/wáter
CO_2_: Carbon dioxide
DMEM/F12: Dulbecco’s modified eagle’s medium/nutrient mixture F-12
DMSO: Dimethyl sulfoxide
FABMS: Fast atom bombardment mass spectrometry
FBS: Fetal bovine serum
gHSQC: Gradient-selected heteronuclear single-quantum correlation; gradient-selected heteronuclear multiple bond correlation
h: Hour
HPLC: High performance liquid chromatography
ID_50_: Median inhibitory dose IC_50_: half inhibitory concentration
L: Liter
LPS: Lipopolysaccharide
Mg: Milligramme
MHz: Megahertz
min: Minute
mL: Milliliter
MTS: 3-(4,5-dimethyl-2-yl)-5-(3-carboxymethoxyphenyl)-2-(4-sulfophenyl)-2H-tetrazolium, inner salt
NaNO_2_: Sodium nitrite
nm: Nanometer
NMR: Nuclear magnetic resonance
NO: Nitric oxide
NOESY: Nuclear Overhauser effect spectroscopy
OD_540_: Optical density at 540 nm
P: Error probability
PGE2: Prostaglandin E2
RP-18e: Reverse phase-18
SD: Standard deviation
TLC: Thin layer chromatography
v/v: Volume/volume
μg: Microgramme
μL: Microliter
μM: Micromolar
